# Numerical Simulation of the Whole Thermal Lensing Process with Z-Scan-Based Methods Using Gaussian Beams

**DOI:** 10.3390/ma14195533

**Published:** 2021-09-24

**Authors:** Georges Boudebs

**Affiliations:** Univ Angers, LPHIA, SFR MATRIX, F-49000 Angers, France; georges.boudebs@univ-angers.fr

**Keywords:** thermal lens, Z-scan, far field diffraction

## Abstract

A general study of the diffracted far field due to thermal lens heating using Gaussian beams is presented. The numerical simulation considers the whole system, including both the optical and the thermal parameters. It is shown that the existing simplified relations found in the literature and used up to now only give the order of magnitude of the thermo-optical coefficients. More accurate, simplified formulas are derived to measure the induced thermal phase shift when working with Z-scan-based methods. Temperature estimation in absorbing media turn out to be more reliable whether using time-resolved or steady-state techniques. The extension of the calculation to the image formation in a 4f system is also addressed.

## 1. Introduction

Many applications use the thermal lens (TL) principle as an ultrasensitive spectrophotometric readout [1] to characterize different physical phenomena, for example (recently found in the literature) to investigate molecular/particle dynamics [2], to measure the photothermal parameters of opaque solids [3], to understand thermal lensing effects using Z-scan-based methods at multiple laser repetition rates and multiple average powers [4,5], to study the effect of highly localized thermal gradients on the catastrophic optical damage process of high-power laser diodes [6], to evaluate optically induced temperature changes in colloidal samples for photothermal therapy [7], to quantify very low concentrations in solutions [8], and to image single light-absorbing nanoparticles by photothermal microscopy [9]. Recently, we have demonstrated experimentally the feasibility of extracting an image of the phase shift induced by TL and applying this method to map an inhomogeneous thin film doped with different concentrations of silver nanoparticles transversally [10]. Since Gordon et al. [11] reported on optical thermal effects, considerable work has been done in this field of a medium acting as a lens to characterize the thermo-optical coefficients of materials. Indeed, the optical energy absorbed in a medium creates a transverse temperature gradient, inducing a variation in the linear index that is commonly called a thermal lens. The first approximation considered a parabolic refractive-index distribution with a Gaussian incident beam [12,13,14]. Then, another step was taken by considering the aberrant nature of the thermal lens and predicting the central intensity variation in the far field of the laser beam in the presence of weakly absorbing media [15,16,17]. Measuring the diffraction of light in a single beam or dual-beam methods [18] allow us to obtain highly sensitive techniques to estimate the material absorbance. Given the complexity of the problem, simplified relationships using physical approximations were derived from theoretical models and used through nonlinear fits to estimate the quantities of interest. These relationships describe the general behavior of the thermal lens qualitatively quite well but are not quantitatively accurate. With the computing power of today’s processors, more accurate calculation can be derived by taking into account the whole system fundamental equations to approach the real nature and behavior of the thermal lens response. 

The first goal of this paper is therefore to extend the field of applicability related to the thermal effect methods in absorbent liquids. More advanced calculation will be obtained showing that the difference can be relatively large when considering the usual rough approximations. Then, we aim to correctly establish a junction between the thermal lens characterization technique and Z-scan-based methods in order to check and improve the reliability of the measurements, particularly in the stationary regime. 

## 2. Theory of the Thermal Effect

The single beam method is considered here, and according to reference [15] some assumptions are made: (i) the thin sample approximation (the beam radius is almost constant along the thickness of the sample); (ii) dimensions of the cell are large compared with the diameter of the beam; (iii) heat conduction through the ends can be neglected, and thus the temperature variation can be taken as purely radial along the x direction. An expression of the temperature change as a function of radius and time ΔT(x,t) can be obtained by solving the non-steady state heat equation appropriate for the problem when a Gaussian beam is illuminating the medium. The following symbols will be used: α absorbance in $m^{-1}$; ρ density in $\mathrm{Kg}/m^{3}$; I beam intensity in $W/m^{2}$; L thickness of the medium (cell) in m; P beam power in W; c specific heat in J/Kg/K; ω beam radius in m; ΔT temperature variation in °K; k thermal conductivity in J/s/m/K; λ wavelength in m.

Taking into account the aberrant nature of the thermal lens [15,17], the variation in temperature in the medium is given by:

(1) $$\Delta T\left(x,t\right)=\frac{2P\alpha }{\pi c\rho \omega^{2}}\int_{0}^{t}\left(\frac{1}{1+2t^{\prime }/t_{c}}\right)\exp\left(\frac{-2x^{2}/\omega^{2}}{1+2t^{\prime }/t_{c}}\right)dt^{\prime },$$

where $t_{c}=\omega^{2}c\rho /4k$ is the characteristic buildup time constant of the thermal lens. This temperature variation induces a change in the linear index according to the following relationship [15]:

(2) $$\Delta n\left(x,t\right)=\frac{dn}{dT}\Delta T\left(x,t\right),$$

with dn/dT denoting the algebraic value of the thermo-optical coefficient, which is often negative, and $\Delta n\left(x,t\right)=n\left(x,t\right)-n_{0}$ where $n_{0}$ is the refractive index at the initial temperature. The phase shift is related to Δn as usual, using Δφ=2πΔnL/λ with λ designing the wavelength of the beam inducing and probing the phase shift.

**Figure 1:**
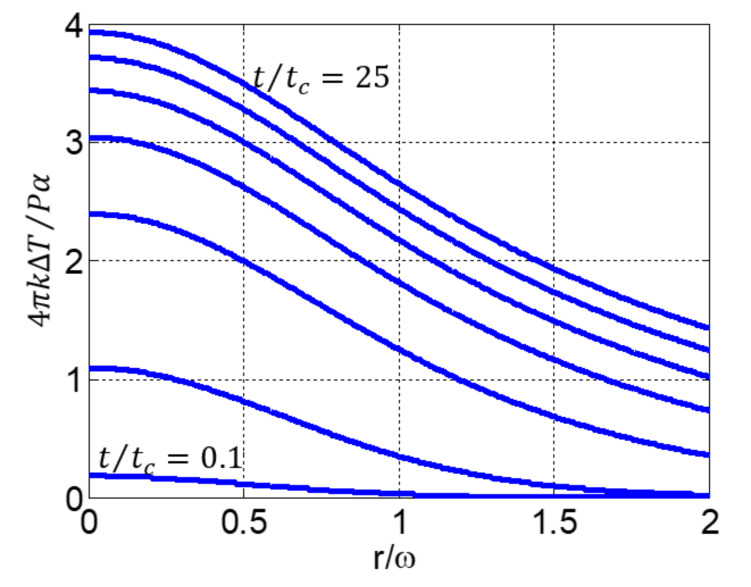
Temperature evolution in the thermal lens at various times (from lowest to highest: $t/t_{c}=0.1,\;1,\;5,\;10,\;15,\;20$ and 25).

**Figure 2:**
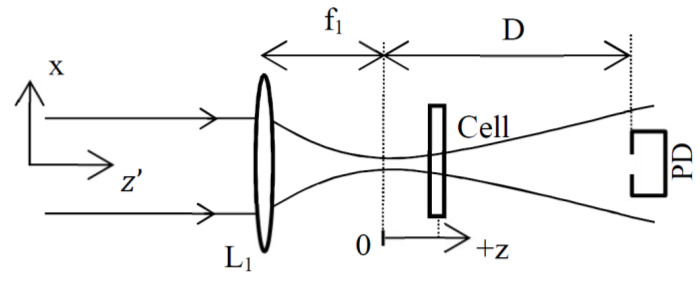
Scheme showing the different positions of the elements. The cell is scanned along the beam direction around the focal plane (*z* = 0). The labels refer to: lens (L_1_) and photodiode (PD).

First, in order to validate our numerical calculations, we present in Figure 1 the temperature distribution in the thermal lens at various times as obtained from Equation (1). The evolution is given according to the same characteristics as those shown in Figure 2 of reference [15]. Indeed, the profiles generated by Equations (1) and (2) are important as they determine the subsequent variation in index and phase due to ΔT, thus the final diffracted field. Note the nonlocal response due to the radial propagation of the heat for increasingly long heating times and the perfect agreement of the result with that found in the later reference. This behavior is understood because at a given point in the profile, we can see that, as $t/t_{c}$ increases, the temperature variation also increases for a given absorbing liquid at a given incident power. Then, the curves tend to tighten, and the temperature variation decreases for large t. Moreover, we can see that the heat propagates transversally beyond the waist of the beam, which reflects a nonlocal response. When x≫ω, the curves continue to decrease and to tend towards 0 since the medium is considered sufficiently large. This approximation is well validated in practice when considering beam-waists of 20–30 µm and cells of about $1\times 10\;cm^{2}$ transverse sections having approximately 1 mm thickness. 

The next step is to understand the influence of this index variation on the diffracted far field of a beam focused into the sample. This approach has been achieved by several authors using more or less severe approximations that give for the most part the intensity at the center of the diffracted beam. Here, we will determine numerically not only the intensity at the system axis for x=0 but also the entire profile of this beam. This will be done without any approximation of the propagation of the wavefront, allowing us to predict the relative variation in the size of the diffracted beam. Then, a comparison will be made between the two results.

## 3. Results and Discussions

As shown in Figure 2, the basic theoretical setup is composed of a focusing lens L_1_, a cell containing the liquid to be tested and a photodiode PD placed in the far field (D≫ω). In our study, it is considered the general case that the cell is scanned along the beam direction around the focal plane (z=0). Therefore, later we can also find results from the simulation related to the Z-scan method [19] and its derivative [20]. Exposure of the sample to the excitation beam is controlled by a chopping wheel placed before L_1_ and not shown in this simplified arrangement. The absorption of the fused silica composing the cell is considered to be negligible and its thickness lower than the Rayleigh range $\left(L<\pi \omega^{2}/\lambda \right)$.

### 3.1. Intensity Profiles Versus z

The diffracted field formation at the output of the setup shown in Figure 2 is described using a numerical model based on Fourier optics (see for details [21,22,23,24]). 

We assume that the electric field at the object focal plane of L_1_ is Gaussian, $E\left(x\right)=E_{0}\exp\left[-x^{2}/\omega_{e}^{2}\right]$, where *x* is the spatial coordinate, $E_{0}$ denotes the on-axis amplitude and $\omega_{e}$ is the beam waist at the entry of the setup. Let $\tilde{S}(u)$ be the spatial spectrum of E: $\tilde{S}\left(u\right)=\tilde{ℱ}\left[E\left(x\right)\right]=\int_{-\infty }^{+\infty }E\left(x\right)\exp\left[-j2\pi \left(ux\right)\right]dx$, where $\tilde{ℱ}$ denotes the Fourier transform operation, *u* is the normalized spatial frequency. In a general way, instead of propagating the field with the Helmholtz equation, we propagate its spectrum over distance $z^{\prime }$ using the transfer function of the wave propagation phenomenon: $H\left(u\right)=\exp\left(j2\pi z^{\prime }\sqrt{1-{\left(\lambda u\right)}^{2}}/\lambda \right)$ [21]. Then, the amplitude of the field at $z^{\prime }$ is obtained by computing the inverse Fourier transform: $E\left(x,\;z^{\prime }\right)={\tilde{ℱ}}^{-1}\left[\tilde{S}\left(u\right)H\left(u\right)\right]$. Additionally, to calculate the output beam after passing through a lens with focal length *f*_1_, we apply the phase transformation related to the thickness variation: $t_{L}\left(x\right)=\exp\left[-j\pi x^{2}/\lambda f_{1}\right]$ [21] in accordance with Figure 2. Then, we propagate the beam up to the sample located at z using $z^{\prime }=f_{1}+z$ in H, the optical transfer function. Next the thermal phase shift related to the response of the material is taken into account using Equation (2) where we define the thermal transmittance as:

(3) $$T_{TL}\left(x,t\right)=\exp\left(j2\pi \Delta nL/\lambda \right),$$

before continuing the propagation, again using H for a distance D−z up to the photodiode. Finally, the intensity $I_{D-z}$ is calculated by squaring the optical field. At this level, we can calculate the characteristics that allow us to trace the induced phase shift in $T_{TL}$ as a function of *z* by calculating the diameter of the output beam or the intensity at the center. 

**Figure 3:**
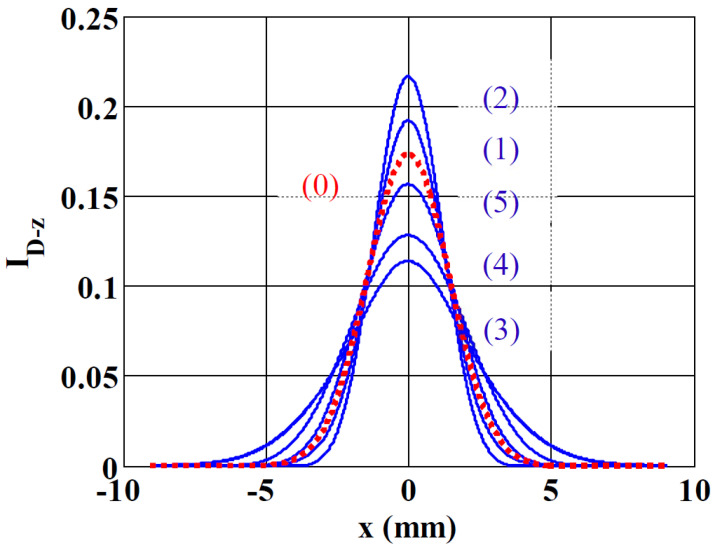
Evolution of the intensity profiles at a distance D=30 cm for *z* varying from −4 mm (1) to +4 mm (5). The other parameters of the simulation are given in the text. The (0) labeled profile (dots) is obtained when there is no TL effect. The y−axis is given with arbitrary units. The numbers labelling the blue solid lines are placed in the order of increasing central intensity profiles.

To illustrate our approach, we have simulated the intensity profile obtained in the steady state regime at a distance D=30 cm from the focal plane of L_1_ having $f_{1}=5.2\;\mathrm{cm}$. The power of the beam, the absorption of the sample, its thickness, the thermo-optical coefficients and the heating time $t_{f}$ are chosen to give a phase shift at the center of the beam in the focus: $\Delta \varphi \left(x=0,z=0\right)=\Delta \varphi_{0}=4.61\;\mathrm{rad}$ for λ=532 nm, where

(4) $$\Delta \varphi_{0}=\frac{4P\alpha L}{\lambda c\rho \omega_{0f}^{2}}\frac{dn}{dT}\int_{0}^{t_{f}}\left(\frac{1}{1+2t^{\prime }/t_{c}}\right)\exp\left(\frac{-2x^{2}/\omega_{0f}^{2}}{1+2t^{\prime }/t_{c}}\right)dt^{\prime },$$

with $\omega_{0f}$ being the beam waist at z=0. The other parameters were defined as follows: $\omega_{e}=0.5\;\mathrm{mm}$, z varies from −4 mm to +4 mm in increments of 2 mm. The intensity profiles obtained from the simulation are shown in Figure 3, where we can see the profile labeled (1) for z=−4 mm, (2) for z=−2 mm, (3) at the bottom of the figure for z=0 mm, (4) for z=2 mm and finally (5) for z=4 mm. The (0) labeled profile is obtained with no thermal lens effect ($\Delta \varphi_{0}=0)$. Its diffracted beam-waist was measured from the simulation profile to be $\omega_{d}=2.8806\;\mathrm{mm}$ while theoretically $\omega_{d}=\lambda D/\pi \omega_{0f}=2.8846\;\mathrm{mm}$. Scanning the cell from negative to positive z values, the peak intensity as shown in profiles (1) and (2) first increases and then suddenly decreases (3), and finally its value increases again in (4) and (5). This is typical behavior of the Z-scan method with negative phase shifts, which show a higher transmittance at pre-focal positions followed by a lower transmittance at post-focal positions [19]. Moreover, at this point note that the relatively high considered phase shift (≈π) is possible when using Helmoltz formalism for the beam propagation (or H, its equivalent in the spectrum domain). No simplifications or approximations are usually made in the literature with diffraction theory (as for example in [15,16,17]) and there are no such stringent conditions on the propagation distance, which can be in the near field. Moreover, we take into account the basic equation describing the effect of the lensing medium (Equation (3)) and not a limited second order expansion assuming a small phase shift $\left(\Delta \varphi_{0}\ll 1\right)$. In addition, the radius of curvature of the spherical wave of the Gaussian beam is often approximated to a parabolic one in the previous references, which limits the analysis of the final intensity to values near the optical axis. In our case, this approximation does not exist and therefore we can study the entire transverse profile of the output beam and accurately measure its diameter with a CCD camera. 

### 3.2. Spatiotemporal Evolution of the Central Output Diffracted Beam

Before analyzing the effects of the above approximations on the final measurement, we will introduce a spatiotemporal image showing the central intensity of the output beam (x=0) when varying z and t, the integration time on the photodiode. The used parameters to generate the results must allow comparison with those of references [15,16,17] in particular to be valid and consistent with the simplifying assumptions. Therefore, let us define θ=PLα(dn/dT)/λk. It is appropriate that this parameter remains sufficiently small because in most experiments invoking thermal lenses using the established analytical relations, θ is of the order of 0.1 or less. Therefore, we considered the parameters shown in Table 1 with t varying from 0 to $t_{f}$ and z from −Z to +Z on 50 and 51 samples, respectively. We also considered the Fresnel reflections on the cell by considering the refractive index of its glass walls $n_{0}$ and we have chosen the size of the beam-waist $\omega_{e}$ corresponding to the output of most CW lasers.

**Figure 4:**
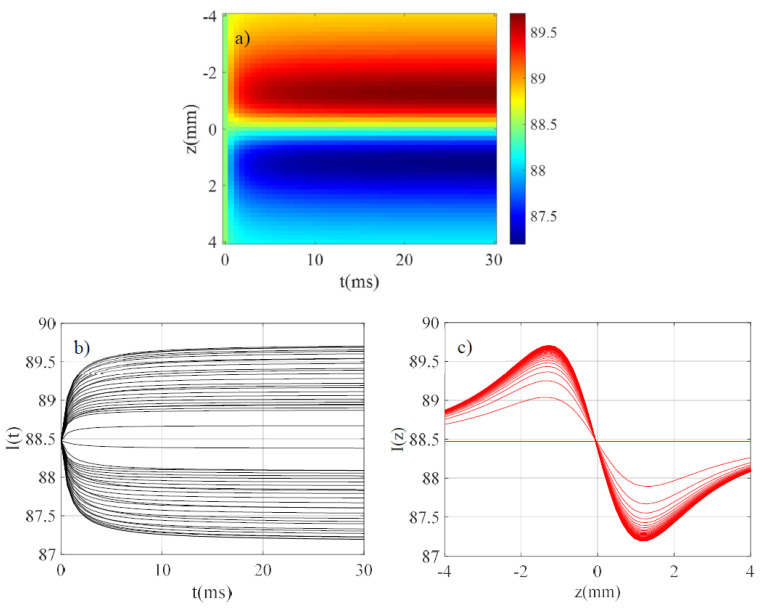
(**a**) Evolution of I(z,t) in arbitrary units; (**b**) evolution of I(t) considering z as a parameter; (**c**) I(z) considering t as a parameter.

These parameters define: θ=−95 mrad, Rayleigh distance $Z_{0}=1.83\;\mathrm{mm}$, $t_{c}=0.77\;\mathrm{ms}$ and the intensity at the focus in the center of the beam $I_{0}=0.591\;\mathrm{MW}/m^{2}$. At the beginning of the program, we define the total number of points so that (i) the Gaussian beam representing the object is sufficiently sampled (10 points for $\omega_{e}$) (ii) the spatial window on which the output beam should be displayed is equal to two times the beam-waist of the larger diffracted beam (obtained at z=0, labeled (3) in Figure 3). These conditions allow us to respect the Nyquist–Shannon sampling theorem and to limit the number of points in order not to unnecessarily lengthen the computation time. In the particular case of the simulation corresponding to Table 1 we had 10,292 sampling points to consider. We can see the result of the calculation in Figure 4a as giving I(z,t) at x=0 in arbitrary units. This image shows the typical characteristics mixing the shapes of the Z-scan traces for a given t and the exponential growth (or decay) for a given z to reach the quasi-stationary regime when $t\gg t_{c}$. Figure 4b shows I(t), the intensity variation versus t considering z as a parameter and Figure 4c shows I(z), the intensity variation versus z considering t as a parameter where one can see clearly what will be considered the “peak” and “valley” characteristics of the Z-scan traces.

### 3.3. Results Comparison 

Let us compare our results with those already established in [15] for low TL regimes, i.e., when *θ* is sufficiently small. Note that the mode-mismatched or matched dual-beam give the same analytical expressions in the particular case of a single beam [16]. In this case, the signal is defined as being the fractional intensity change when the cell is located at z after t times exposure of the material: S(z,t)=[I(z,t)−I(z,∞)]/I(z,∞). Analytical calculations for the in-axis far field intensity variation provided [15]:

(5) $$S\left(z,t\right)=-1+\frac{1-\theta tan^{-1}\left[\frac{2V}{3+V^{2}+\left(9+V^{2}\right)\left(\frac{t_{c}}{t}\right)}\right]}{1-\theta tan^{-1}\left[\frac{2V}{3+V^{2}}\right]},$$

where $V=z/Z_{0}$ with $Z_{0}=\pi \omega_{0f}^{2}/\lambda$. 

Setting t=0 in Equation (5) allows us to obtain the total fractional intensity change as a function of V:

(6) $$S\left(V\right)=-1+\frac{1}{1-\theta tan^{-1}\left(\frac{2V}{3+V^{2}}\right)}$$

The derivative of Equation (6) allows us to optimize this signal, finding positions of the sample at $V=\pm \sqrt{3}$ where S is extremal. Moreover, it is possible to get $t_{c}$ the characteristic time of the absorbing liquid letting $z=\sqrt{3}Z_{0}$ in Equation (5) where the signal becomes:

(7) $$S\left(t\right)=-1+\frac{1-\theta tan^{-1}\left(\frac{0.577}{1+t_{c}/t}\right)}{1-\theta \left(0.577\right)}$$

**Figure 5:**
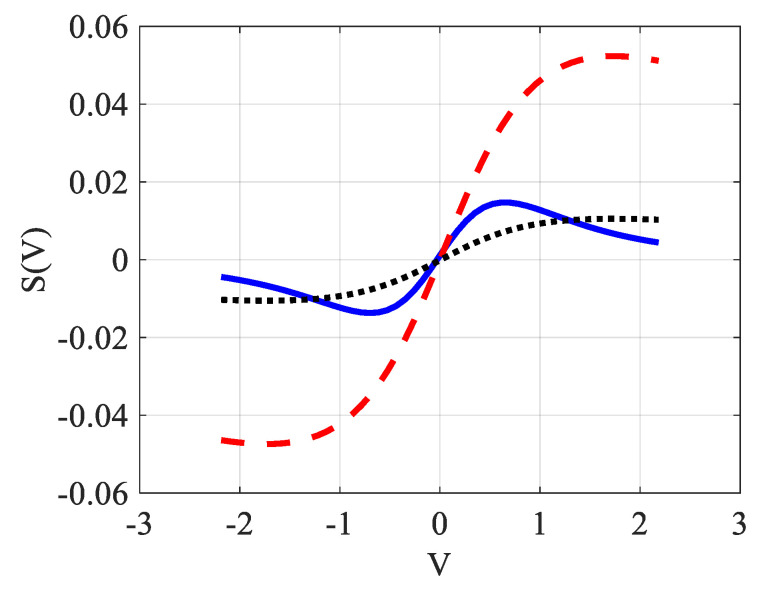
(color online): Comparison of the signal given by the simulation without approximations (solid blue line) with that from Equation (6) (dashed red line) used to characterize the TL effect. The dotted (black) line is the fitting of the solid blue line representing the exact signal.

Equations (6) and (7) will be tested next, comparing their results with those given by the exact previous simulation, whose parameters appear in Table 1. Figure 5 shows the evolution of S(V) as given by Equation (6) in the dashed (red) points while the simulated exact evolution for the same parameters should be given by the curve as it appears in the solid (blue) line. In other words, if we assimilate the exact-computed acquisition signal to the solid (blue) line, the fitting defined by Equation (6) and shown in the dotted (black) line estimates θ as 4.7 times smaller. Recall that this signal is defined as S(z,0)=[I(z,0)−I(z,∞)]⁄I(z,∞), which represents the relative change in intensity as a function of z from the initial heating time up to steady state.

We also notice that the maximum and minimum of the signal given by the solid blue line (obtained without resorting to approximations) and the analytical function defined in Equation (6) do not give the same values. Indeed, as already mentioned, Equation (6) is obtained following a rather rough calculation, so it is not surprising to see that its extremums also do not correspond exactly to the correct values that are obtained around V≈±0.7, separated therefore by a distance $\approx \sqrt{2}$. 

**Figure 6:**
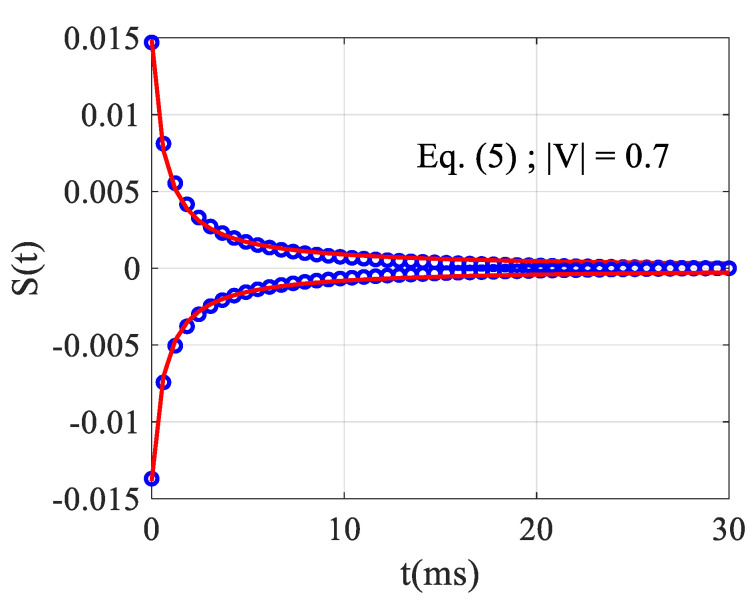
Nonlinear fit (solid red lines) of *S*(*t*) showing the temporal evolution of the exact numerical data (empty blue circles) characterizing the rise and the fall time of the TL effect.

We can now study Equation (7) or its analogous Equation (5) using the correct values for the maximum and the minimum. We have used a nonlinear least squares routine to fit the temporal behavior at these positions obtained with the exact numerical simulation as if they were experimental acquisitions. At the minimum, for V=−0.7, we obtained θ=−36.9 mrad and $t_{c}=0.50\;\mathrm{ms}$, while at the maximum, for V=0.61, we obtained θ=−42.2 mrad and $t_{c}=0.50\;\mathrm{ms}$. It should be noted that a small difference is observed in the fitting results when moving from negative to positive z because the response S(t) is not symmetrical (see Figure 6 for t=0). This asymmetrical response is much more pronounced for higher absolute values of θ. In any case, the mean measured value is given as: θ=−39.5 mrad and $t_{c}=0.5\;\mathrm{ms}$ which always underestimates the correct values (−95 ms and 0.77 ms, respectively) with relative errors of 60% on θ and 35% on $t_{c}$. 

Comparing the results given by the fits according to Equation (5) on one side and Equation (6) on the other, one can notice that we do not obtain the same value for θ. Indeed, the fit realized in Figure 5 by Equation (6) takes into account the average value of the signal along the entire length of the V axis. Therefore, for V<1.2, a part of the fitting curve (dashed line) is situated below the curve to fit (solid line) and for higher V it is the opposite. While in Figure 6, the fit done by Equation (5) is considered at the extremums of S, which should logically give a slightly higher value for θ—and this is indeed the case. 

Note that the sensitivity of the photodiode for different incident wavelengths should not affect the signal because it is always calculated as being the relative variation of the intensity. Regarding the bandwidth, the characteristic responses of the liquids are of the order of a millisecond, which does not represent any particular technical difficulty for a photodiode and for the oscilloscope to follow the rise or fall of the signal according to time”.

### 3.4. Simplified Relations Using Z-scan-based Methods

**Figure 7:**
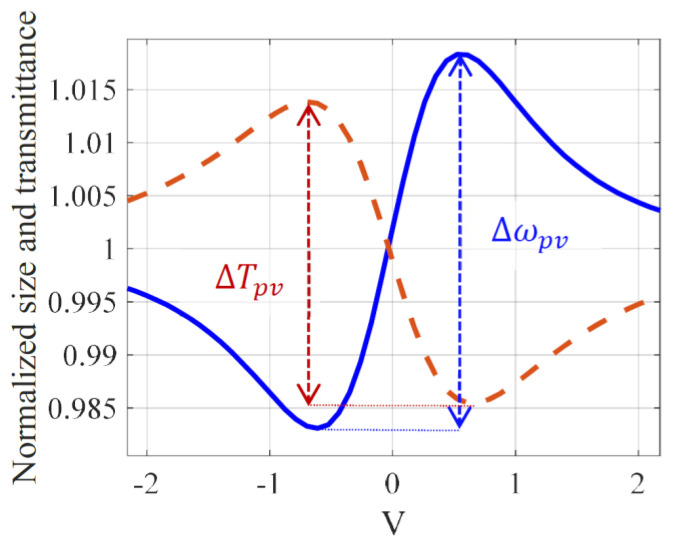
(color online): The relative size variation in solid (blue) line and the normalized Z-scan transmittance in dashed (red) line versus the position of the cell. The blue curve is shifted to 1.

We will end this analysis by providing the relationship between the induced phase shift and the signal it generates in a single beam stationary mode. As already shown in the 4f setup [20], we can then measure the variation of the beam size at the output of an imaging system or directly, following the scheme in Figure 2. Indeed, the 4f setup acts through its second lens, forming the image as a far field diffraction, provided that $D\gg \;Z_{0}$. In the presence of a Gaussian beam, we checked that the relations obtained for the relative size variation of the output beam would be the same with or without this second lens. Therefore, to minimize the numerical calculation, we will present the results relative to the simplified scheme in Figure 2, knowing that the same relations would be obtained in the 4f system. The parameters of Table 1 will always be considered. Figure 7 shows the numerical simulation of the Z-scan trace, in the dashed (red) line, along with the relative size variation of the beam, in the solid (blue) line, measured using the D4σ method as in [20]. Based on the calculation of the second order moment of the diffracted beam, the D4σ method gives four times the standard deviation of the intensity profile distribution. The difference between the peak and the valley is a linear function of the induced phase shift as already shown experimentally in [10] using a CCD at the output of a 4f system. Using D4σ method we found: $\Delta \omega_{pv}=0.17\Delta \varphi_{0}$ with $\Delta \omega_{pv}=\left(\omega_{TL}-\omega_{d}\right)/\omega_{d}$ where $\omega_{TL}$ represents the size of the beam in the presence of the TL and $\omega_{d}$ without, i.e., in the low TL regime. Furthermore, the relation obtained for Z-scan linking the signal to the induced phase shift in the center of the beam at z=0 (with a closed aperture sensing the maximum of the intensity profile) is $\Delta T_{pv}=0.14\Delta \varphi_{0}$. The sensitivity is defined as the ratio of the output of a measurement system with respect to an input. The coefficient 0.14 found here is in good agreement with that found in the literature. According to [25], the sensitivity of Z-scan in the steady-state regime is 0.15.

One could notice that the sensitivity of D4σ and Z-scan methods are almost the same. D4σ measurement has already demonstrated a sensitivity similar to that of Z-scan by characterizing the Kerr-type optical nonlinearities [22]. The main advantage of D4σ comes from the fact that the signal to noise ratio is much higher given the use of all the pixels defining the spatial extension of the beam on the CCD. This is to be compared with the few pixels used when processing the measurement relative to the linear transmittance of the closed aperture located at the top hat of the diffracted beam. Moreover, the obtained steady-state signals with a CCD are not sensitive to the characteristic time for the transient induced nonlinear index variation because the CCD takes a longer time to acquire an image. In addition, a study is being developed to propagate vortex beams [26] in this imaging system, which could improve the sensitivity of the measurement by increasing the contrast in the image of the phase object that would be placed at the entrance of the setup. This numerical model has been widely used with third order optical nonlinearities induced by high pulsed laser intensities in the picosecond regime. Here, the same propagation model is used (for more details, see [20,22,23,24]), but what is new is the use of a thermal lens phase shift in the focal region instead of that relative to third order optical nonlinearities.

In summary, the use of rough approximations to obtain simplified analytical formulas is no longer necessary with today’s computing power. It would be necessary to calibrate the setup with known parameters that can be measured beforehand in a classical way. A more accurate determination of the thermo-optical coefficients is then possible. On the other hand, many publications have appeared with large values of the nonlinear third order optical index due to thermal effects generated by either a relatively low intensity CW laser, relatively long pulse duration in the nanosecond regime or high repetition rate in the femtosecond regime. We need to study the exact influence of these parameters on the generated signals. This is an area that deserves full attention and efforts to refine the nonlinear measurements where many existing artifacts are found in this field. Therefore, this paper paves the way to study from a metrological point of view the phase shift induced by the thermal lens effect that comes sometimes with the instantaneous Kerr induced ones as was found initially in [27] and more recently in [5]. 

## 4. Conclusions

A numerical analysis method has been developed using the transfer function of the spectrum propagation to calculate the near-field diffraction beam. A study of the simplified analytical relations generally used to evaluate the thermo-optical coefficients in a single mode beam has been established using the output of a classical CW laser. These analytical rough relations have the advantage of existing but only give an order of magnitude of the desired coefficients to be measured: θ is underestimated with 60% relative error although the characteristic time $t_{c}$ remains acceptable with 35% error. Other characteristics of the signal can be misleading, such as its position of the minimum and the maximum, which are found to be separated by $∼\sqrt{2}Z_{0}$ instead of $2\sqrt{3}Z_{0}$. Finally, a study of the Z-scan-based method sensitivities has yielded relationships that may be useful for attaining more accurate measurements to determine the steady-state thermal lens phase shift more precisely.

## Figures and Tables

**Table 1:** The considered parameters used for the simulation.

$\alpha \left(m^{-1}\right)$	λ (nm)	L (mm)	$f_{1}\;\left(cm\right)$	$\omega_{e}\;\left(mm\right)$	P (mW)	D (cm)	$n_{0}$	$t_{f}\;\left(ms\right)$	Z (mm)
90	532	1	5.2	0.5	0.3	30	1.5	30	4

## Data Availability

The data presented in this study are available on request from the corresponding author.
